# 560 Assessment of Ointment Containing β-sitosterol in Healing of Burns in Children up to 5 Years

**DOI:** 10.1093/jbcr/iraf019.189

**Published:** 2025-04-01

**Authors:** Liang Wei, Mahmoud Sakr

**Affiliations:** MEBO International; Alexandria University

## Abstract

**Introduction:**

To assess the efficacy and safety of burn regenerative therapy (BRT) using ointment containing β-sitosterol in healing of second- and third-degree burns in children.

**Methods:**

The study population included 20 consecutive burned children up to 5 years, admitted with 2nd- and/or 3rd-degree burns, up to 20% TBSA. 11 girls and 9 boys (age 2.66 ± 1.73 years). All patients were treated with β-sitosterol ointment dressing every 8 hours. Burn wound surface moist swabs were taken from all children at weekly intervals and tissue biopsy was taken from deep burns only. The following was recorded during hospital say; sepsis, wound discharge, time of eschar separation and formation of granulation tissue, healing rate, need of skin graft, drug reactions and duration of hospital stay. During follow-up (6 months) assessment included final scar quality and appearance, deformity, graft take, and organ function.

**Results:**

Scalds were the cause of burn in 15 victims (75%). The face was involved in 7 children (35%). The mean TBSA was 10.8 ± 4.74 % and 30% of burns were 3rd-degree (deep) burns. The mean time of eschar separation was 6.253.02 days, and of healthy granulation tissue appearance was 14.63.65 days. Superficial 2nd-degree burns healed within one week with normal skin elasticity, no scar formation and no obvious pigmentation. Deep 2nd-degree burns healed within 2-3 weeks with normal skin in 18 children. Skin grafting (with 100% take) was required in children with 3rd-degree burns only. Hospital stay was 12.10 5.13 days. No disturbance of function or deformity was encountered. The commonest organisms cultured were Pseudomonas Aeruginosa and MRSA, alone or in combination.

**Conclusions:**

(1) all 2nd-degree burns heal spontaneously with normal skin (2) good debridement, rapid eschar liquefaction and early granulation tissue formation in 3rd-degree burns is achieved, with complete take of skin grafts, (3) there is a definite anti-microbial action, effective against resistant organism, and (4) ointment containing β-sitosterol is safe, easy to apply, keeping a physiological moist environment and supplying indispensable nutritional substrates and enzymes necessary for physiological wound healing.

**Applicability of Research to Practice:**

Burn regenerative therapy (BRT) using ointment containing β-sitosterol can be used to treat second- and third-degree burns in children with significant clinical efficacy.

**Funding for the Study:**

N/A

